# Bis(2-chloro­benzoato-κ*O*)bis­(1-vinyl­imidazole-κ*N* ^3^)copper(II)

**DOI:** 10.1107/S1600536808030237

**Published:** 2008-09-24

**Authors:** Juan Zhao

**Affiliations:** a College of Mechanical Engineering, Qingdao Technological University, Qingdao 266033, People’s Republic of China

## Abstract

In the title compound, [Cu(C_7_H_4_ClO_2_)_2_(C_5_H_6_N_2_)_2_], each Cu^II^ ion, located on an inversion center, has a slightly distorted square-planar coordination geometry formed by two 1-vinyl­imidazole mol­ecules [Cu—N = 1.954 (6) Å] and two 2-chloro­benzoate anions [Cu—O = 1.958 (6) Å]. Weak inter­molecular C—H⋯O hydrogen bonds contribute to the crystal packing stability.

## Related literature

A square-planar coordination environment of Cu^II^ was also observed in bis­(3-hydroxy­benzoato-κ*O*)bis­(1*H*-imidazole-κ*N* ^3^)copper(II), see: Liu *et al.* (2006 ▶).
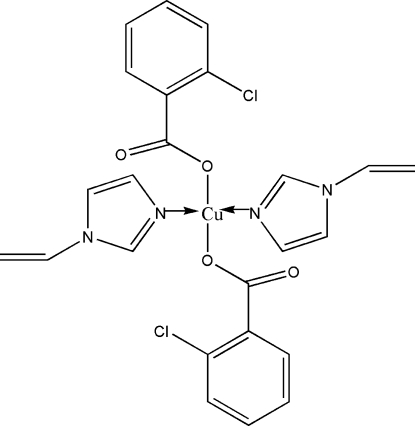


## Experimental

### 

#### Crystal data

[Cu(C_7_H_4_ClO_2_)_2_(C_5_H_6_N_2_)_2_]
*M* *_r_* = 562.89
Monoclinic, 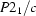

*a* = 7.9360 (16) Å
*b* = 11.236 (2) Å
*c* = 14.190 (3) Å
β = 104.36 (3)°
*V* = 1225.8 (5) Å^3^
*Z* = 2
Mo *K*α radiation
μ = 1.15 mm^−1^
*T* = 293 (2) K
0.20 × 0.10 × 0.10 mm

#### Data collection

Bruker SMART 1K CCD area-detector diffractometer
Absorption correction: multi-scan (*SADABS*; Sheldrick, 2004 ▶) *T*_min_ = 0.803, *T*_max_ = 0.894
2204 measured reflections
2115 independent reflections
1620 reflections with *I* > 2σ(*I*)
*R*_int_ = 0.039

#### Refinement

*R*[*F* ^2^ > 2σ(*F* ^2^)] = 0.073
*wR*(*F* ^2^) = 0.192
*S* = 1.04
2115 reflections
154 parameters
49 restraints
H-atom parameters constrained
Δρ_max_ = 0.73 e Å^−3^
Δρ_min_ = −0.89 e Å^−3^

### 

Data collection: *SMART* (Bruker, 2001 ▶); cell refinement: *SAINT* (Bruker, 2001 ▶); data reduction: *SAINT*; program(s) used to solve structure: *SHELXTL* (Sheldrick, 2008 ▶); program(s) used to refine structure: *SHELXTL*; molecular graphics: *SHELXTL*; software used to prepare material for publication: *SHELXTL* and local programs.

## Supplementary Material

Crystal structure: contains datablocks global, I. DOI: 10.1107/S1600536808030237/cv2449sup1.cif

Structure factors: contains datablocks I. DOI: 10.1107/S1600536808030237/cv2449Isup2.hkl

Additional supplementary materials:  crystallographic information; 3D view; checkCIF report

## Figures and Tables

**Table 1:** Hydrogen-bond geometry (Å, °)

*D*—H⋯*A*	*D*—H	H⋯*A*	*D*⋯*A*	*D*—H⋯*A*
C1—H1*A*⋯O2^i^	0.93	2.56	3.484 (10)	174
C3—H3*A*⋯O1^ii^	0.93	2.49	2.918 (8)	108
C5—H5*A*⋯O2^i^	0.93	2.45	3.342 (9)	160
C11—H11*A*⋯O2^iii^	0.93	2.60	3.460 (9)	155

Symmetry codes: (i) 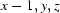; (ii) 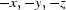; (iii) 
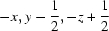
.

**Fig. 1.**
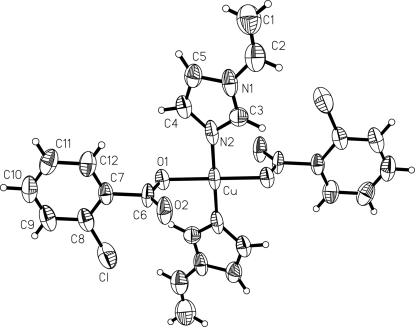
The molecular structure of (I), showing 50% probability displacement ellipsoids and the atom-numbering scheme. The unlabelled atoms are related with the labelled ones by symmetry operation (-x, -y, -z).

## supplementary crystallographic information

### Comment

In the title compound, (I) (Fig. 1), each Cu ion is coordinated by a pair of 1-vinylimidazole ligands and a pair of monodentate carboxylate groups, affording a square planar N_2_O_2_ coordination geometry. The CuN_2_O_2_ core involving the central atoms is almost perfectly square planar. The *trans* angles are all 180° for symmetry requirements and the *cis* ones are 89.52 (19)° and 90.48 (19)° for N—Cu—O, respectively. The Cu—*N*(imidazole) distance is 1.954 (6)Å and The Cu—O bond distance is 1.958 (4) Å. These bond distances are comparable with the reported data (Liu *et al.*, 2006). The five atoms of CuN_2_O_2_ are coplanar. Distances and angles in 1-vinylimidazole are normal. The weak intermolecular C—H···O interactions (Table 1) stabilize the structure.

### Experimental

Copper(II) acetate hydrate(2.00 g, 10 mmol), 1-vinylimidazole(0.99 g, 10 mmol) and 2-chlorobenzoic acid(1.55 g, 10 mmol) were dissolved in water(40 ml). The pH of the solution was adjusted to 7 with 0.2*M* sodium hydroxide. The solution was filtered; blue single crystals of (I) were isolated after several days.

### Refinement

H atoms were positioned geometrically (C—H = 0.93 Å) and allowed to ride on their parent atoms with *U*_iso_(H) = 1.2 *U*_eq_(C).

### Crystal data

**Table:**

[Cu(C_7_H_4_ClO_2_)_2_(C_5_H_6_N_2_)_2_]	*F*(000) = 574
*M**_r_* = 562.89	*D*_x_ = 1.525 Mg m^−^^3^
Monoclinic, *P*2_1_/*c*	Mo *K*α radiation, λ = 0.71073 Å
Hall symbol: -P 2ybc	Cell parameters from 25 reflections
*a* = 7.9360 (16) Å	θ = 10–14°
*b* = 11.236 (2) Å	µ = 1.15 mm^−^^1^
*c* = 14.190 (3) Å	*T* = 293 K
β = 104.36 (3)°	Block, blue
*V* = 1225.8 (5) Å^3^	0.20 × 0.10 × 0.10 mm
*Z* = 2	

### Data collection

**Table:**

Bruker SMART 1K CCD area-detector diffractometer	2115 independent reflections
Radiation source: fine-focus sealed tube	1620 reflections with *I* > 2σ(*I*)
graphite	*R*_int_ = 0.039
Thin–slice ω scans	θ_max_ = 25.2°, θ_min_ = 2.3°
Absorption correction: multi-scan (*SADABS*; Sheldrick, 2004)	*h* = −9→9
*T*_min_ = 0.803, *T*_max_ = 0.894	*k* = 0→13
2204 measured reflections	*l* = 0→16

### Refinement

**Table:**

Refinement on *F*^2^	Primary atom site location: structure-invariant direct methods
Least-squares matrix: full	Secondary atom site location: difference Fourier map
*R*[*F*^2^ > 2σ(*F*^2^)] = 0.073	Hydrogen site location: inferred from neighbouring sites
*wR*(*F*^2^) = 0.192	H-atom parameters constrained
*S* = 1.04	*w* = 1/[σ^2^(*F*_o_^2^) + (0.07*P*)^2^ + 6*P*] where *P* = (*F*_o_^2^ + 2*F*_c_^2^)/3
2115 reflections	(Δ/σ)_max_ < 0.001
154 parameters	Δρ_max_ = 0.73 e Å^−^^3^
49 restraints	Δρ_min_ = −0.89 e Å^−^^3^

### Special details

**Table:**

Geometry. All e.s.d.'s (except the e.s.d. in the dihedral angle between two l.s. planes) are estimated using the full covariance matrix. The cell e.s.d.'s are taken into account individually in the estimation of e.s.d.'s in distances, angles and torsion angles; correlations between e.s.d.'s in cell parameters are only used when they are defined by crystal symmetry. An approximate (isotropic) treatment of cell e.s.d.'s is used for estimating e.s.d.'s involving l.s. planes.
Refinement. Refinement of *F*^2^ against ALL reflections. The weighted *R*-factor *wR* and goodness of fit *S* are based on *F*^2^, conventional *R*-factors *R* are based on *F*, with *F* set to zero for negative *F*^2^. The threshold expression of *F*^2^ > σ(*F*^2^) is used only for calculating *R*-factors(gt) *etc*. and is not relevant to the choice of reflections for refinement. *R*-factors based on *F*^2^ are statistically about twice as large as those based on *F*, and *R*- factors based on ALL data will be even larger.

### Fractional atomic coordinates and isotropic or equivalent isotropic displacement parameters (Å^2^)

**Table:**

	*x*	*y*	*z*	*U*_iso_*/*U*_eq_	
Cu	0.0000	0.0000	0.0000	0.0439 (4)	
Cl	0.3736 (3)	−0.36078 (18)	0.02395 (17)	0.0794 (6)	
O1	0.0066 (5)	−0.1740 (3)	0.0077 (3)	0.0455 (10)	
N1	−0.3796 (8)	0.0695 (5)	0.1425 (4)	0.0550 (14)	
C1	−0.6165 (13)	0.1373 (8)	0.2115 (6)	0.088 (3)	
H1A	−0.6754	0.0655	0.1961	0.105*	
H1B	−0.6638	0.1972	0.2422	0.105*	
O2	0.1925 (7)	−0.1406 (4)	0.1509 (3)	0.0647 (14)	
N2	−0.1911 (7)	0.0008 (4)	0.0642 (4)	0.0527 (14)	
C2	−0.4664 (11)	0.1540 (7)	0.1896 (5)	0.066 (2)	
H2A	−0.4120	0.2270	0.2063	0.079*	
C3	−0.2436 (8)	0.0933 (6)	0.1068 (5)	0.047	
H3A	−0.1907	0.1676	0.1114	0.057*	
C4	−0.3049 (9)	−0.0881 (6)	0.0741 (5)	0.0504 (15)	
H4A	−0.3010	−0.1654	0.0513	0.060*	
C5	−0.4234 (9)	−0.0489 (6)	0.1212 (5)	0.0537 (16)	
H5A	−0.5138	−0.0917	0.1360	0.064*	
C6	0.1057 (9)	−0.2068 (5)	0.0889 (5)	0.0501 (16)	
C7	0.1031 (8)	−0.3403 (5)	0.1088 (4)	0.0433 (13)	
C8	0.2177 (9)	−0.4153 (6)	0.0813 (4)	0.0513 (15)	
C9	0.2146 (11)	−0.5370 (6)	0.0999 (6)	0.0662 (19)	
H9A	0.2926	−0.5882	0.0812	0.079*	
C10	0.0945 (11)	−0.5801 (6)	0.1461 (6)	0.0676 (19)	
H10A	0.0906	−0.6613	0.1580	0.081*	
C11	−0.0196 (11)	−0.5057 (7)	0.1749 (5)	0.0648 (18)	
H11A	−0.0990	−0.5360	0.2071	0.078*	
C12	−0.0162 (10)	−0.3853 (6)	0.1558 (5)	0.0580 (17)	
H12A	−0.0943	−0.3344	0.1746	0.070*	

### Atomic displacement parameters (Å^2^)

**Table:**

	*U*^11^	*U*^22^	*U*^33^	*U*^12^	*U*^13^	*U*^23^
Cu	0.0696 (7)	0.0204 (5)	0.0350 (5)	0.0026 (5)	0.0001 (5)	0.0014 (4)
Cl	0.1031 (15)	0.0558 (11)	0.0838 (14)	0.0230 (11)	0.0319 (12)	0.0061 (10)
O1	0.060 (2)	0.030 (2)	0.043 (2)	0.0052 (19)	0.0066 (19)	0.0033 (18)
N1	0.088 (4)	0.033 (3)	0.035 (3)	0.009 (3)	−0.004 (3)	0.002 (2)
C1	0.115 (7)	0.071 (6)	0.079 (6)	0.004 (5)	0.029 (6)	−0.011 (5)
O2	0.094 (4)	0.029 (2)	0.058 (3)	−0.003 (2)	−0.006 (3)	−0.009 (2)
N2	0.075 (3)	0.024 (2)	0.048 (3)	0.008 (3)	−0.008 (3)	0.001 (2)
C2	0.096 (6)	0.054 (5)	0.042 (4)	0.004 (4)	0.004 (4)	0.007 (3)
C3	0.047	0.047	0.047	0.000	0.012	0.000
C4	0.068 (4)	0.035 (3)	0.043 (3)	0.008 (3)	0.006 (3)	0.003 (3)
C5	0.067 (4)	0.043 (3)	0.045 (4)	−0.001 (3)	0.001 (3)	0.009 (3)
C6	0.074 (4)	0.020 (3)	0.050 (4)	−0.004 (3)	0.004 (3)	0.000 (3)
C7	0.064 (3)	0.028 (3)	0.031 (3)	0.002 (2)	−0.001 (2)	−0.002 (2)
C8	0.072 (4)	0.037 (3)	0.040 (3)	0.011 (3)	0.004 (3)	−0.002 (3)
C9	0.091 (5)	0.039 (3)	0.062 (4)	0.017 (3)	0.008 (4)	−0.001 (3)
C10	0.091 (5)	0.035 (3)	0.063 (4)	−0.005 (3)	−0.007 (3)	0.007 (3)
C11	0.086 (4)	0.053 (4)	0.051 (4)	−0.017 (3)	0.010 (3)	0.013 (3)
C12	0.085 (4)	0.042 (3)	0.047 (3)	−0.002 (3)	0.017 (3)	0.007 (3)

### Geometric parameters (Å, °)

**Table:**

Cu—N2^i^	1.954 (6)	C3—H3A	0.9300
Cu—N2	1.954 (6)	C4—C5	1.356 (9)
Cu—O1^i^	1.958 (4)	C4—H4A	0.9300
Cu—O1	1.958 (4)	C5—H5A	0.9300
Cl—C8	1.751 (7)	C6—C7	1.528 (8)
O1—C6	1.278 (7)	C7—C8	1.366 (8)
N1—C3	1.327 (8)	C7—C12	1.383 (9)
N1—C5	1.389 (9)	C8—C9	1.394 (10)
N1—C2	1.433 (9)	C9—C10	1.372 (11)
C1—C2	1.317 (10)	C9—H9A	0.9300
C1—H1A	0.9300	C10—C11	1.367 (11)
C1—H1B	0.9300	C10—H10A	0.9300
O2—C6	1.225 (7)	C11—C12	1.381 (9)
N2—C3	1.320 (8)	C11—H11A	0.9300
N2—C4	1.377 (8)	C12—H12A	0.9300
C2—H2A	0.9300		
			
N2^i^—Cu—N2	180.0 (3)	C4—C5—N1	104.5 (6)
N2^i^—Cu—O1^i^	89.52 (19)	C4—C5—H5A	127.7
N2—Cu—O1^i^	90.48 (19)	N1—C5—H5A	127.7
N2^i^—Cu—O1	90.48 (19)	O2—C6—O1	125.6 (5)
N2—Cu—O1	89.52 (19)	O2—C6—C7	119.7 (6)
O1^i^—Cu—O1	180.0 (4)	O1—C6—C7	114.6 (5)
C6—O1—Cu	109.9 (4)	C8—C7—C12	119.8 (6)
C3—N1—C5	107.1 (6)	C8—C7—C6	120.9 (6)
C3—N1—C2	125.1 (6)	C12—C7—C6	119.4 (6)
C5—N1—C2	127.8 (6)	C7—C8—C9	120.4 (7)
C2—C1—H1A	120.0	C7—C8—Cl	120.9 (5)
C2—C1—H1B	120.0	C9—C8—Cl	118.6 (5)
H1A—C1—H1B	120.0	C10—C9—C8	118.9 (7)
C3—N2—C4	103.6 (6)	C10—C9—H9A	120.5
C3—N2—Cu	125.9 (4)	C8—C9—H9A	120.5
C4—N2—Cu	130.4 (4)	C11—C10—C9	121.1 (7)
C1—C2—N1	125.6 (8)	C11—C10—H10A	119.4
C1—C2—H2A	117.2	C9—C10—H10A	119.4
N1—C2—H2A	117.2	C10—C11—C12	119.6 (7)
N2—C3—N1	113.2 (6)	C10—C11—H11A	120.2
N2—C3—H3A	123.4	C12—C11—H11A	120.2
N1—C3—H3A	123.4	C11—C12—C7	120.1 (7)
C5—C4—N2	111.6 (6)	C11—C12—H12A	119.9
C5—C4—H4A	124.2	C7—C12—H12A	119.9
N2—C4—H4A	124.2		
			
N2^i^—Cu—O1—C6	92.5 (4)	Cu—O1—C6—O2	−4.0 (9)
N2—Cu—O1—C6	−87.5 (4)	Cu—O1—C6—C7	172.6 (4)
O1^i^—Cu—N2—C3	−17.5 (5)	O2—C6—C7—C8	−92.3 (8)
O1—Cu—N2—C3	162.5 (5)	O1—C6—C7—C8	90.9 (7)
O1^i^—Cu—N2—C4	160.1 (5)	O2—C6—C7—C12	87.2 (8)
O1—Cu—N2—C4	−19.9 (5)	O1—C6—C7—C12	−89.6 (7)
C3—N1—C2—C1	168.6 (8)	C12—C7—C8—C9	0.4 (10)
C5—N1—C2—C1	−8.3 (11)	C6—C7—C8—C9	179.9 (6)
C4—N2—C3—N1	0.2 (7)	C12—C7—C8—Cl	−178.6 (5)
Cu—N2—C3—N1	178.3 (4)	C6—C7—C8—Cl	0.9 (8)
C5—N1—C3—N2	−0.6 (7)	C7—C8—C9—C10	0.1 (10)
C2—N1—C3—N2	−178.0 (5)	Cl—C8—C9—C10	179.0 (6)
C3—N2—C4—C5	0.3 (7)	C8—C9—C10—C11	−0.8 (11)
Cu—N2—C4—C5	−177.7 (4)	C9—C10—C11—C12	1.1 (11)
N2—C4—C5—N1	−0.7 (7)	C10—C11—C12—C7	−0.7 (11)
C3—N1—C5—C4	0.8 (7)	C8—C7—C12—C11	0.0 (10)
C2—N1—C5—C4	178.0 (6)	C6—C7—C12—C11	−179.6 (6)

Symmetry codes: (i) −*x*, −*y*, −*z*.

### Hydrogen-bond geometry (Å, °)

**Table:**

*D*—H···*A*	*D*—H	H···*A*	*D*···*A*	*D*—H···*A*
C1—H1A···O2^ii^	0.93	2.56	3.484 (10)	174
C3—H3A···O1^iii^	0.93	2.49	2.918 (8)	108
C5—H5A···O2^ii^	0.93	2.45	3.342 (9)	160
C11—H11A···O2^iv^	0.93	2.60	3.460 (9)	155

Symmetry codes: (ii) *x*−1, *y*, *z*; (iii) −*x*, −*y*, −*z*; (iv) −*x*, *y*−1/2, −*z*+1/2.
